# Effect of flanged diffuser divergence angle on wind turbine: A numerical investigation

**DOI:** 10.1371/journal.pone.0287053

**Published:** 2023-06-15

**Authors:** Tahir Abbas Jauhar, Muhammad Imtiaz Hussain, Tayybah Kiren, Waseem Arif, Sajjad Miran, Gwi Hyun Lee

**Affiliations:** 1 Department of Mechanical Engineering, University of Gujrat, Gujrat, Pakistan; 2 Agriculture and Life Sciences Research Institute, Kangwon National University, Chuncheon, Korea; 3 Department of Computer Science (RCET), University of Engineering and Technology Lahore, Lahore, Pakistan; 4 Interdisciplinary Program in Smart Agriculture, Kangwon National University, Chuncheon, Korea; NUST: National University of Sciences and Technology, PAKISTAN

## Abstract

Power augmentation in a small-scale horizontal axis wind turbine, with its rotor encased in a flanged diffuser is explored. The power output of the wind turbine varies with changes in the diffuser design and the resulting back pressure. Reduction in this back pressure also results in early flow separation at the diffuser surface, which hinders turbine performance. The main aim of this study is to numerically investigate the local configuration of the wind turbine location inside the diffuser by varying diffuser angles and wind speeds. Therefore, shroud and flange were modeled and analyzed using the computational fluid dynamic (CFD) analyses and experiments were performed at two wind speeds 6 *m/s* and 8 *m/s* with and without the diffuser for model validation. The divergence angle of 4° was found to have no flow separation, thus maximizing flow rate. The proposed design shows wind speed improvement of up to 1.68 times compared to the baseline configuration. The corresponding optimum flange height was found to be 250 *mm*. However, increasing the divergence angle had a similar output. The dimensionless location of wind turbine was found to be between 0.45 and 0.5 for 2° and 4° divergence angle respectively. Furthermore, the maximum augmentation location varies with wind speed and diffuser’s divergence angle as described by dimensionless location of wind turbine, thus presenting a noteworthy contribution to the horizontal axis wind turbine area with the flanged diffuser.

## Introduction

Energy is the necessity of life. Human beings have been converting energy into power to get the work done. The methods devised for energy harvest led to a rapid depletion of fossil fuels. Energy harvesting methods have also adversely affected the climate giving rise to global warming over the years. The energy wastage by transmission losses leads to a net increase in energy requirements. Micro power generation technologies should be used to overcome these transmission losses. However, micro grids require high capital investment with no performance forecasts. These micro grids, if made affordable, could be as small as a house. The options available for micro grid generation are hydropower, photovoltaic power, and wind power. These energy harvesting methods are environmentally friendly and sustainable. Renewability in domestic power generation is expected to reduce the World’s *CO*_2_ emissions by a large number. The hydropower is limited to those areas with access to a substantial water head to convert potential energy to electrical energy. Photovoltaic power is available only during the day, whereas wind power depends on wind flow. Wind power generation does not emit harmful gases or radioactive waste. Wind power can be harvested during the day as well as at night using a wind turbine. It is plentiful in most regions of the earth. The provision of electric power from renewable energy resources is counted as one of the factors of industrialization and economic growth of any nation [1].

Samrat et al. discuss the importance of energy for the socioeconomic development of developing countries like Malaysia, particularly in the electrification of islands and remote rural areas [2]. It proposes a battery storage hybrid standalone photovoltaic-wind energy power supply system that integrates different green energy sources and power electronic converters to overcome the unpredictability of renewable energy sources and maintain a constant DC-link voltage for the load. Simulation results suggest that the proposed system can work under variable weather and load conditions. Work has been on predictions models to predict the adoptions of renewable wind energy in developing countries [3]. The article by Barbosa et al. describes a study that calculates the feasibility and cost of implementing a 100% renewable energy-based power system in South and Central America by 2030, using an hourly resolved energy model [4]. The study finds that renewable energy sources can meet the region’s electricity demand and generate electricity for water desalination and synthetic natural gas production, and that using existing hydro dams as virtual batteries can reduce the need for additional storage technologies. Additionally, integrating water desalination and power-to-gas into the system can lead to a reduction in cost and electricity generation.

**Fig 1:**
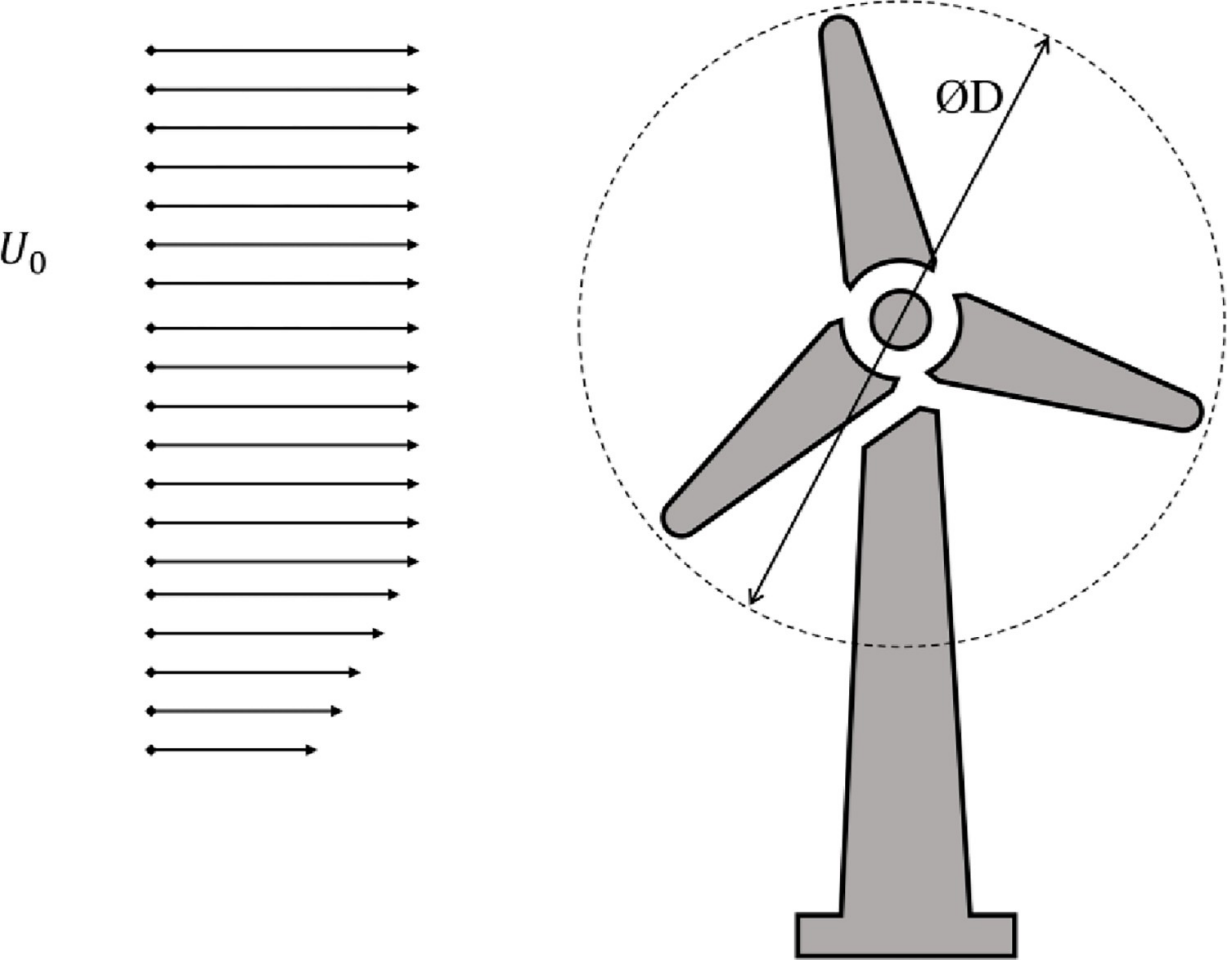
Free-stream velocity and rotor diameter.

In wind power generation, mechanical energy is produced when wind flows through the turbine rotor; as shown in Fig 1, “D” represents the rotor’s diameter and “*U*_0_” is the free stream velocity of the wind. Wind power is related to the mass flow rate of the wind. Wind velocity is related to wind power by a factor of a cube. With an increase in the wind velocity by two, the wind power will be increased by eight. Therefore, increasing the air velocity locally in the wind turbine’s vicinity will increase the power at lower global air velocities. The wind speed effects the RE, thus affecting the design of the wind turbine [5]. The air velocity can be locally increased using a diffuser effect. The mass flow rate in a shrouded wind turbine increases due to sub-atmospheric pressure at the diffuser’s exit and supports power augmentation.

Oman et al. [6] presented a case study on the cost-effective diffuser for wind power augmentation by experimentally confirming the technical feasibility. Controlled expansion in the diffuser creates significant sub-atmospheric pressure. In the Diffuser Augmented Wind Turbine (DAWT) case, power augmentation was 4.24 times compared to conventional turbines [7]. The geometry of the diffuser affects the mass flow rate. Ohya et al. [8] showed that exit pressure could be further lowered by adding flange/brim at the exit of a diffuser by induced vortices. More air is drawn due to a decrease in the backpressure. Adding brim to the diffuser structure significantly increases the structure’s weight while simultaneously lowering the sub-atmospheric pressure, Ohya et al. [9] developed a modified compact brim, which reduced the size of the diffuser but had the same effect. Tip losses are reduced in diffuser augmented wind turbines [9, 10]. Hansen et al. [11] reported that the Betz Limit could be exceeded concerning the relative increase in the mass flow rate ratio. Van Bussel [12] presented the momentum theory explaining the role of negative back pressure on the wind turbine’s performance. The experimental study for flanged diffuser wind turbines was carried out [13]. The experimental studies of diffuser angle and length reported an increase in generated power from 1.6 to 2.1 times that of without diffuser [14].

It was concluded by applying momentum theory to various wind energy concentrator systems that considerable improvement in wind speed was made by a shroud only [15]. Moreover, the comparison was made between a shrouded wind turbine and a bare wind turbine using an actuator disk model in CFD based on the axial momentum’s theoretical expression. The comparison proved that the shrouded wind turbine had a higher power output [11]. The power augmentation can be explained through experimental work conducted on a shrouded wind turbine with 3 m rotor diameter with length to diameter and inlet to exit ratios as 2.67 and 4 respectively. The power coefficient was 1.40 at a wind speed of 5m/s based on the rotor diameter. The flow separation that occurred in the wind tunnel was responsible for this performance improvement [9]. The review of experimental studies in shrouded wind turbines has been reported to achieve power augmentation of up to five times [16].

Werle and Presz [17] presented a simple analytical wind or water turbine model based on the classical fluid dynamic theories. Theoretically, this model concluded that the turbine with duct has more power than the bare turbine. To develop a cost-effective, compact diffuser for HAWT using axial momentum theory, the exit to inlet area ratio has been assumed as 2.75 as a baseline design point. The reduction in this baseline design point led to a reduction in power augmentation factor with a relatively lower cost and vice versa. It was concluded that DAWT is an economically viable concept for a small wind turbine if light fiberglass is used to fabricate the diffuser [18]. The optimization of linearized diffuser for shrouded wind turbines is presented to achieve the power coefficient higher than the Betz limit [19]. Alkhabbaz et al. [20] studied the impact of compact diffuser shroud on wind turbine. Shrouded invelox wind collector design was studied and reported wind speed increase from 1.52 to 1.68 times based on the height of buildings [21]. Bontempo et. al. investigated various parameters of diffuser through the design of experiment methodology [22]. The simplex algorithm has also been used to perform the diffuser optimization [23].

Thermo-economic study of the HAWT shroud by Al-Sulaiman et al. [24] with three distinct shroud area ratios (outlet to throat area) that were 1.5, 1.3 and 1.1. The result showed that to some extent (without flow separation), increasing the shroud area ratio improves turbine efficiency and decreases the cost of power generated. The study also shows that the cost of power produced decreases as high wind speeds occur. In the same background, 2 years later, Al-Sulaiman et al. [25] performed an exergoeconomic study of an ejector-augmented shrouded HAWT, depending on the ejector inlet area ratio.

The central part of the study focused on ratio of the area at the ejector inlet side to the area at the exit of the primary shroud with three distinct values on the rate of power generation and the cost of its output. The result was shown to increase the power generated and decrease the cost of generated power by raising the ejector inlet area ratio [25]. In the discussion on using ducts to increase wind velocity in wind turbines, Kumar [26] concluded that the ducted turbine rotor could achieve a high-power coefficient value that has crossed the Betz limit. However, the duct design should be configured locally and globally according to different flow conditions.

The field of diffuser augmented wind turbines has received significant attention in published research. However, the placement of the wind turbine within the diffuser in relation to changes in flow velocities remains an underexplored area. This study aims to address this knowledge gap by conducting a numerical investigation into the effect of varying diffuser angles and wind speeds on the optimal location of the wind turbine within the diffuser. This investigation represents a novel contribution to the field and has the potential to improve the efficiency and effectiveness of diffuser augmented wind turbines.

## Experimental methodology

The rotor’s diameter is one meter. The total diameter becomes 1.25 *m* by incorporating the area of flanges. The wind tunnel available in the Fluid Mechanics lab of the Pakistan Institute of Engineering and Science has a diameter of 0.8 *m*. Thus, it is not suitable for providing a uniform flow for our experimental setup. The wind turbine was mounted on a Suzuki pickup truck following the approach [27], and the truck was driven at fixed speeds to obtain wind flow due to relative motion between the wind turbine and wind. The relative speed was measured using an anemometer, and then it was maintained at the constant speed during the experiment.

**Fig 2:**
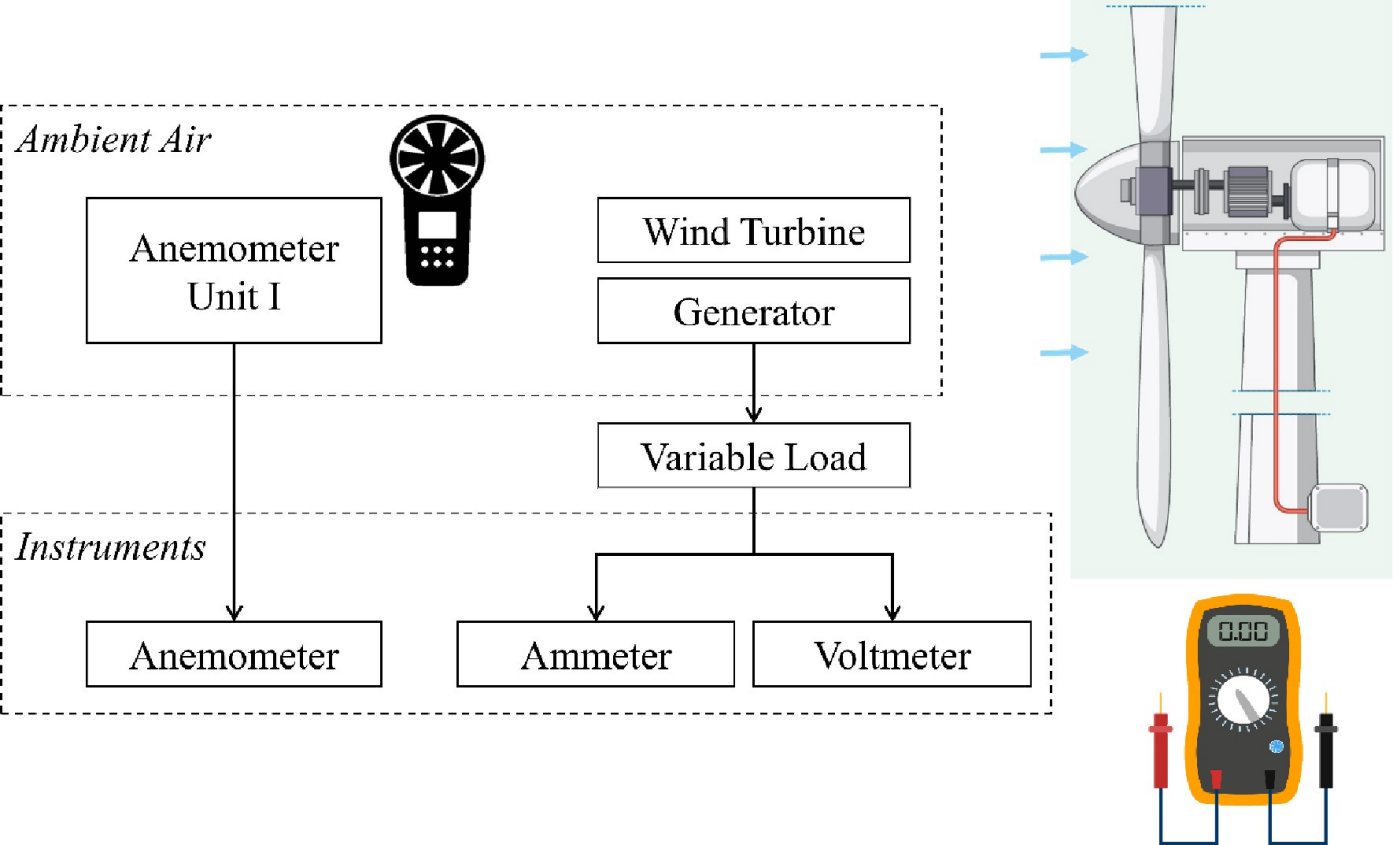
Schematic diagram of experimental setup.

The schematic diagram of experimental setup is shown in Fig 2. The diagram shows two zones, one is ambient air zone and the second is instrumentation zone. In the ambient air zone, the anemometer measures the flow speed and wind turbine assembly along with generator converts kinetic energy of air into electrical energy. A variable load is connected with the generator. In the instrumentation zone, there is the second portion of anemometer to report the flow speed and ammeter and voltmeter measure the current and voltage respectively. Therefore, when anemometer reports 6 m/s and 8 m/s, the measurements of current and voltage are taken by varying the load from 1 Ω to 200 Ω.

The experiment was conducted by varying the load at the wind turbine generator for the flow velocities of 6 *m*/*s* and 8 *m*/*s*. A total of eight experiments were performed. Four experiments were performed with a diffuser and four without the diffuser. There were two wing configurations, three-wing and six-wing. The four experiments without the diffuser are tabulated in Table 1. In Table 2, four experiments with the diffuser are tabulated.

**Table 1:** Power output without diffuser.

*U*_0_	6 m/s		8 m/s	
**Wings**	3	6	3	6
**Load (Ω)**	P = VI (W)	P = VI (W)	P = VI (W)	P = VI (W)
**1**	16	25	4	25
**3**	5.88	21.3	8.3	16.3
**5**	4.05	20	12.8	33.8
**10**	6.4	14.4	32.4	22.5
**20**	5	9.8	20	20
**30**	16.1	7.5	26.1	16.1
**50**	16.82	5.12	24.5	12.5
**100**	8.41	2.89	16	6.25
**200**	4.5	2	6.48	4.5

**Table 2:** Power output with diffuser.

*U*_0_	6 m/s		8 m/s	
**Wings**	3	6	3	6
**Load (Ω)**	P = VI (W)	P = VI (W)	P = VI (W)	P = VI (W)
**1**	1.7	4.8	16.0	9.0
**3**	3.0	8.3	8.3	33.3
**5**	20.0	12.8	20.0	28.8
**10**	14.4	16.9	40.0	16.9
**20**	14.5	20.0	28.8	39.2
**30**	4.8	10.8	32.0	24.3
**50**	32.0	13.5	35.3	24.5
**100**	17.6	3.6	18.5	13.7
**200**	6.1	2.6	9.7	8.0

## Numerical methodology

All numerical simulations were carried out by solving the Navier-Stokes (N-S) [28] Eqs for an incompressible fluid. The governing Eqs (1–3) are used for 2D unsteady fluid flows.


$$\frac{\partial u}{\partial x}+\frac{\partial v}{\partial y}=0$$ (1)

$$\frac{\partial u}{\partial t}+\frac{\partial u^{2}}{\partial x}+\frac{\partial uv}{\partial y}=-\frac{\partial P}{\partial x}+\frac{1}{Re}(\frac{\partial^{2}u}{\partial x^{2}}+\frac{\partial^{2}u}{\partial y^{2}})$$ (2)

$$\frac{\partial u}{\partial t}+\frac{\partial uv}{\partial x}+\frac{\partial v^{2}}{\partial y}=-\frac{\partial P}{\partial y}+\frac{1}{Re}(\frac{\partial^{2}v}{\partial x^{2}}+\frac{\partial^{2}v}{\partial y^{2}})$$ (3)

Where *u* and *v* are the non-dimensional components of the velocity vector in the *x* and *y* directions respectively, *P* is non-dimensional static pressure, and *t* is non-dimensional time.

### Governing equations

Shear Stress Transport (SST) model has the same advantages as a regular k–ω. Under adverse pressure gradients, the SST model accounts for turbulent shear stress transport and makes highly precise estimates of the onset and volume of flow separation. For boundary layer simulations with high precision, SST is recommended. Compared to regular k-ω, this is less optimal for free shear flows due to the dependence on wall distance. Mesh resolution close to the wall is needed. The Reynolds Stress model may be more suitable for flows with abrupt increases in strain rate or spinning flows, whereas the SST model may be more appropriate for separated flows. In the free-stream, the SST formulation moves to a k-ε behavior, avoiding the usual k-problem of the model being too responsive to the inlet free-stream turbulence properties. Therefore, SST k-ω [29] model is selected. The following Eqs (4) and (5) were discretized and solved using ANSYS Fluent.


$$\frac{\partial (\rho k)}{\partial t}+\frac{\partial (\rho u_{i}k)}{\partial x_{j}}=P-\beta^{*}\rho \omega k+\frac{\partial }{\partial x_{j}}[(\mu +\sigma_{k}\mu_{t})\frac{\partial k}{\partial x_{j}}]$$ (4)

Specific Dissipation Rate:

$$\frac{\partial (\rho \omega )}{\partial t}+\frac{\partial (\rho u_{j}\omega )}{\partial x_{j}}=\frac{\gamma }{\vartheta_{t}}P-\beta \rho \omega^{2}+\frac{\partial }{\partial x_{j}}\left[\left(\mu +\sigma_{\omega }\mu_{t}\right)\frac{\partial \omega }{\partial x_{j}}\right]+2(1-F_{1})\frac{\rho \sigma_{\omega^{2}}}{\omega }\frac{\partial k}{{\partial x}_{j}}\frac{\partial \omega }{{\partial x}_{j}}$$ (5)

### Boundary conditions

Inlet is defined as velocity inlet for the boundary conditions, whereas outlet is defined as pressure outlet. Diffuser wall and flange are defined as no slip. A fan boundary condition is defined at the diffuser throat and axis-symmetric transient analysis is performed.

### Domain and grid study

**Fig 3:**
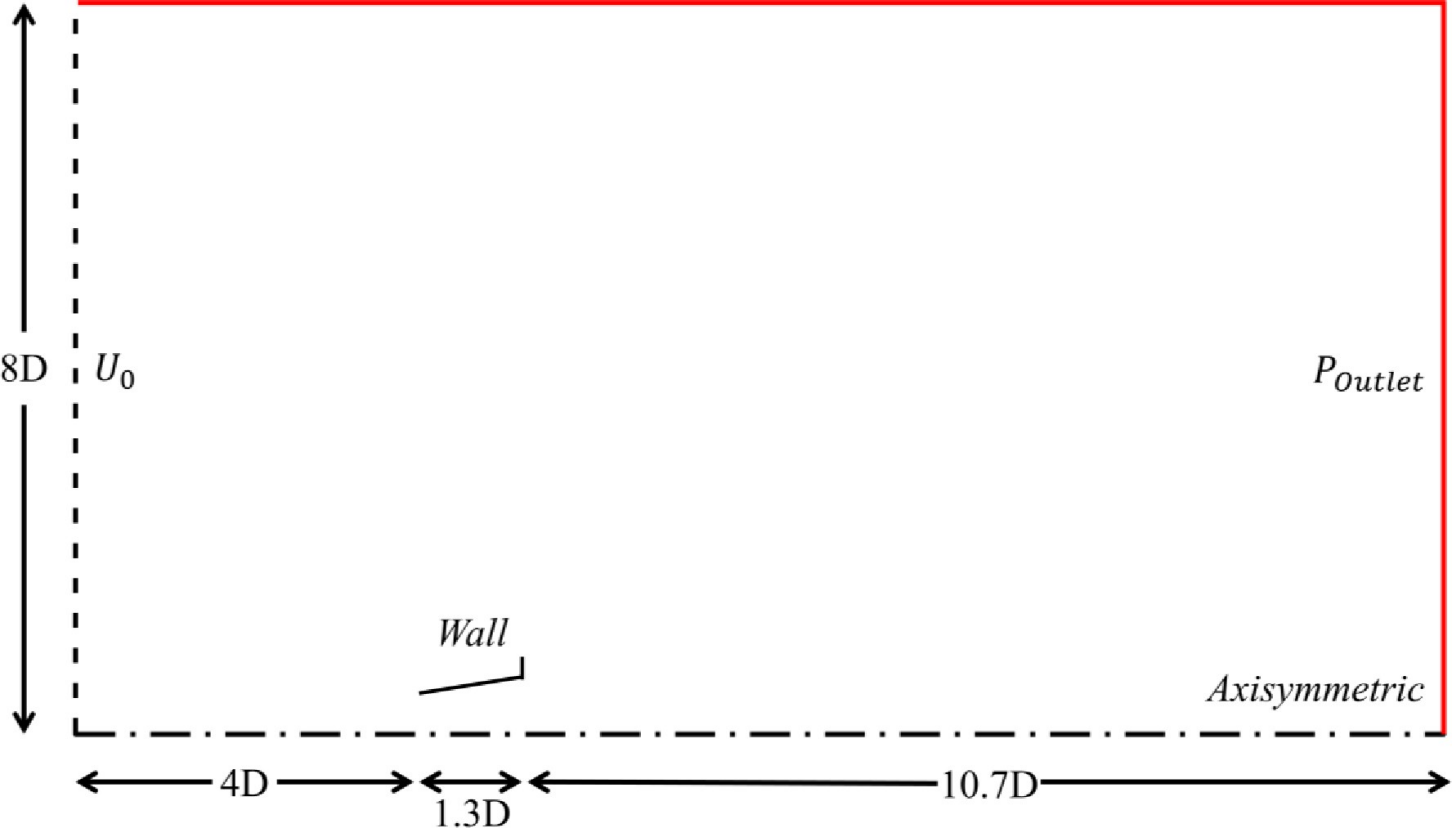
Boundary conditions.

**Fig 4:**
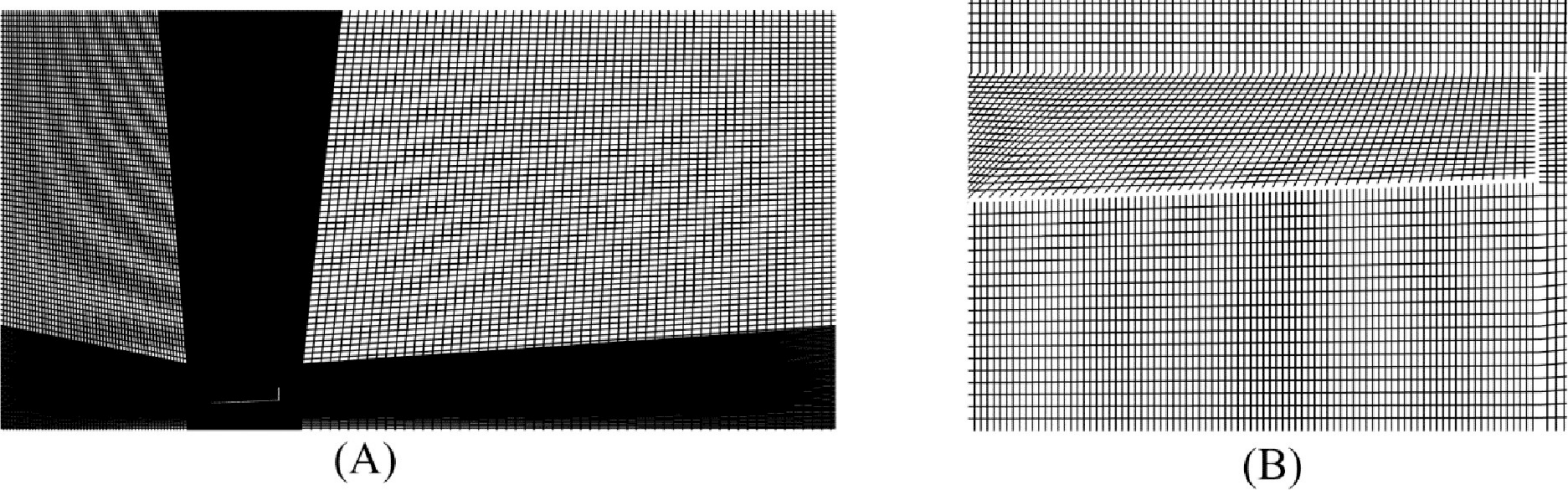
**(A)** Whole Computational Domain **(B)** Diffuser Domain.

The governing equations were discretized using computational software ANSYS FLUENT [30] based on the finite volume code. Fig 3 shows the whole computational domain with boundary conditions. The upstream diffuser domain is 4D and the downstream domain is 12D. The height of the domain is 8D. Thus, computational domain comprises of −4⩽x/D⩽12 and 0⩽y/D⩽8 as shown in Fig 3. The mesh was created using the multi-block technique. The refined mesh was generated near the diffuser body, as shown in Fig 4. A fine O type mesh was used to capture the separating layers accurately at the diffuser and brim tail end. A C-type mesh was used for the diffuser, as shown in Fig 4(B), and a coarse mesh was distributed for the rest of the flow field Fig 4(A).

The reported domain has been validated by the domain independence study. The variations in velocity at the diffuser throat were stopped when overall domain height is 8D, upstream length is 4D and downstream length is 12D. Thus, depicting domain independence.

The number of divisions on edges of multiple blocks was parameterized against the average maximum velocity at the diffuser throat for grid independence study. Grid independence was achieved. A grid size that consists of 32335 elements was chosen for this study. Mesh independence has been tabulated in Table 3, where the change in average velocity at the throat and *Y*^*+*^ value at flange have been reported against nodes and elements. The flow regime under consideration is turbulent; therefore, an adaptive time step study has been performed for time independence study. For the grid independence study, the CFL-based adaptive time step was used with a total time of 25s for 6 m/s inlet velocity. The total time chosen was 25s based on the inlet velocity and total flow regime. The step size was found to be 0.00083, where the solution was convergent.

**Table 3:** Mesh independence study.

Nodes	Elements	*Y* ^*+*^	U_throat_avg_
**11357**	11094	~10	8.0393
**20482**	20121	~2.5	7.9480
**25771**	25357	~1.50	7.9151
**32801**	**32335**	**~0.95**	**7.8899**
**35019**	34543	~0.45	7.8877

### Diffuser parameter optimization

The two factors controlling the diffuser’s performance are the divergence angle of the diffuser and the flange height in the current study. In the Design Modeler of Ansys, both divergence angle and height of flange were parameterized sequentially. Initially, the height of the flange was taken as h = 0.2 m, and the divergence angle was calculated while keeping the rest of the parameter’s constant, as shown in Table 4. Then, the height of the flange was chosen arbitrarily to induce the effects of flange height in the diffuser. The average velocity at the throat was used as the monitoring parameter. The maximum flow speed was achieved using the α = 4°. After the optimization, the divergence angle of the flange height was optimized. The optimized flange height was h = 0.25 m, as shown in Table 5. Finally, the diffuser was manufactured with the obtained dimensions, and the model validation study was performed. The model validation study has been explained in the next section.

**Table 4:** Divergence angle study at h = 0.2 m.

α	U_throat_avg_
**10**	8.3
**9**	8.7
**8**	8.0
**7**	7.8
**6**	7.4
**5**	7.2
*4*	*9*.*2*
**3**	9.1
**2**	6.5

**Table 5:** Flange height study at α = 4°.

h	U_throat_avg_
**0.02**	7.0
**0.1**	6.2
**0.15**	7.0
**0.2**	9.2
*0*.*25*	*9*.*4*
**0.3**	7.0

### Model validation

The electrical power calculated from the experiments and the percentage increase in efficiency can be described by comparing electrical power with computational power.

The power present in the wind can be given by the following Eq 6.


$$P=\frac{1}{2}{\rho U}^{3}A$$ (6)

Where:

*A* = area of the cross-section that depends upon the diameter.

*U* = velocity of the wind

*ρ* = density of air

According to Betz Limit, an ideal type of turbine can extract only 59.3% of the wind energy [5, 31] which depicts in Eq 7.


$$P=0.59\frac{1}{2}{\rho U}^{3}A$$ (7)

For an ideal case, the coefficient of power is 0.59, which varies from 0.35 to 0.45 for good designs. The blade profiles used in this study were based on the study performed by Nongdhar et al. [27, 32]. Electrical power was obtained experimentally, whereas computational power is based on an equation where 0.35 factor is used instead of 0.59.

### Experimental validation

The percentage efficiency reported in the Table 6 indicates a decrease in wind turbine efficiency with increased wind velocity. These differences are similar when a comparison is performed to increase velocity for “with” and “without” diffuser cases. Wind speed augmentation is reported in Table 7 with computational power augmentation. “Power Augmentation (Experimental)” is referred to as electrical power. The inefficiencies of transmission and generator were not included in the “Power Augmentation (Computational).” Therefore, the computational model is validated from Tables 6 and 7. The difference in the P_exp_ and P_num_ can be further explained based on the computation flow behavior within the diffuser versus the actual fluid behavior. The diffuser behaves as a single pipe; however, the fluid regime converts into a tubular channel in actual flow. This is because of the wind turbine’s hub, thus decreasing the overall velocity augmentation. Further computational analysis are performed to show the effect of α, where α = 2⁰, 4⁰ and 7⁰. In addition, the inlet wind velocities were varied from U_0_ = 2 m/s to U_0_ = 10 m/s with increments of 2 m/s.

**Table 6:** Wind power with and without diffuser.

U_0_	Diffuser	P_exp_	U_throat_avg_	P_num_	ⴄ
**6**	Without	16.82	6.00	36.37	46.25
**6**	with	32.00	8.40	99.79	32.06
**8**	Without	33.8	8	86.20	39.21
**8**	with	40	13.2	387.24	10.33

**Table 7:** Power augmentation.

U_0_	Wind Speed Augmentation	Power Augmentation (Experimental)	Power Augmentation (Computational)
**6**	1.4	1.90	5.21
**8**	1.65	1.183	4.50

## Results and discussions

**Fig 5:**
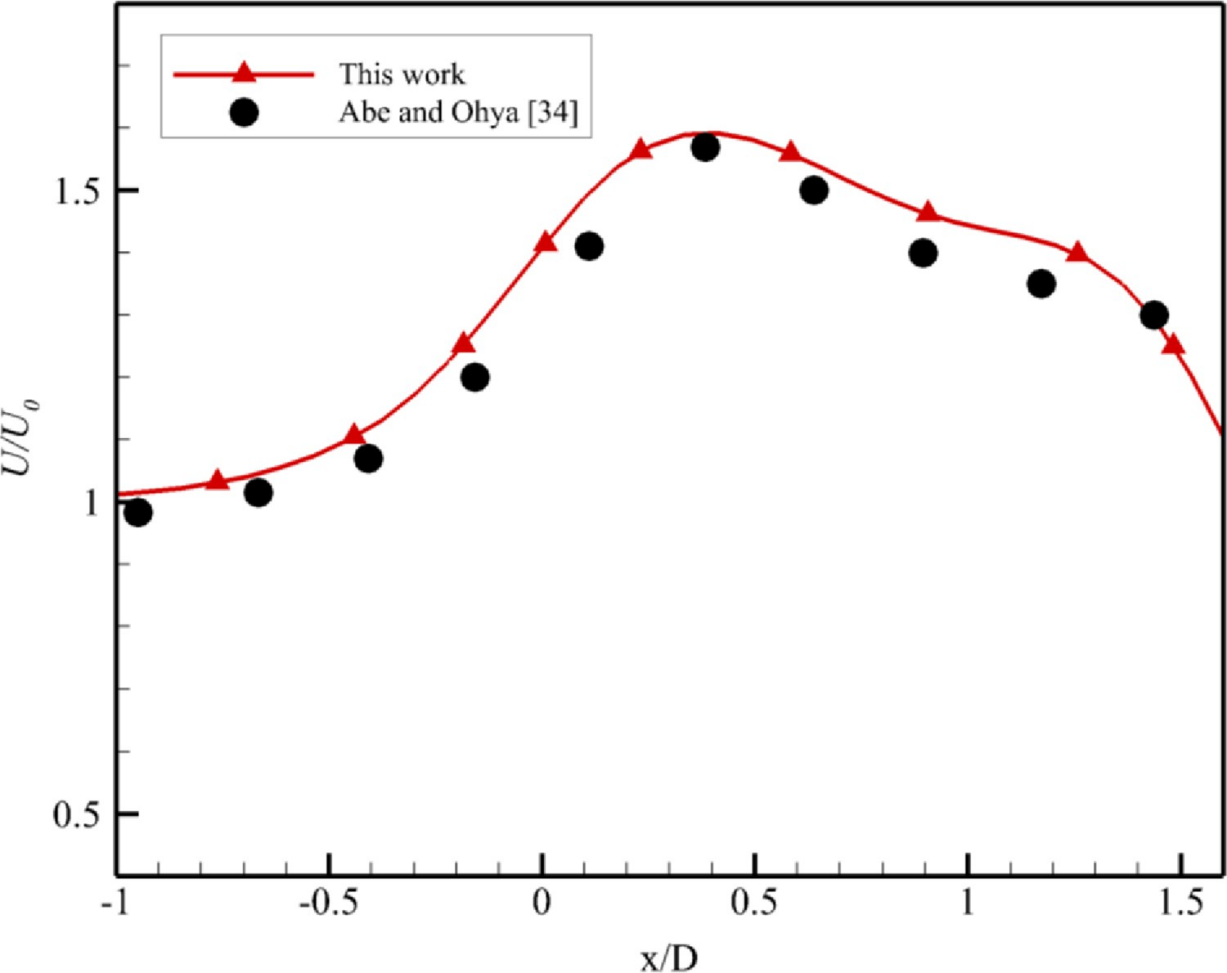
Comparison with experimental work [34].

**Fig 6:**
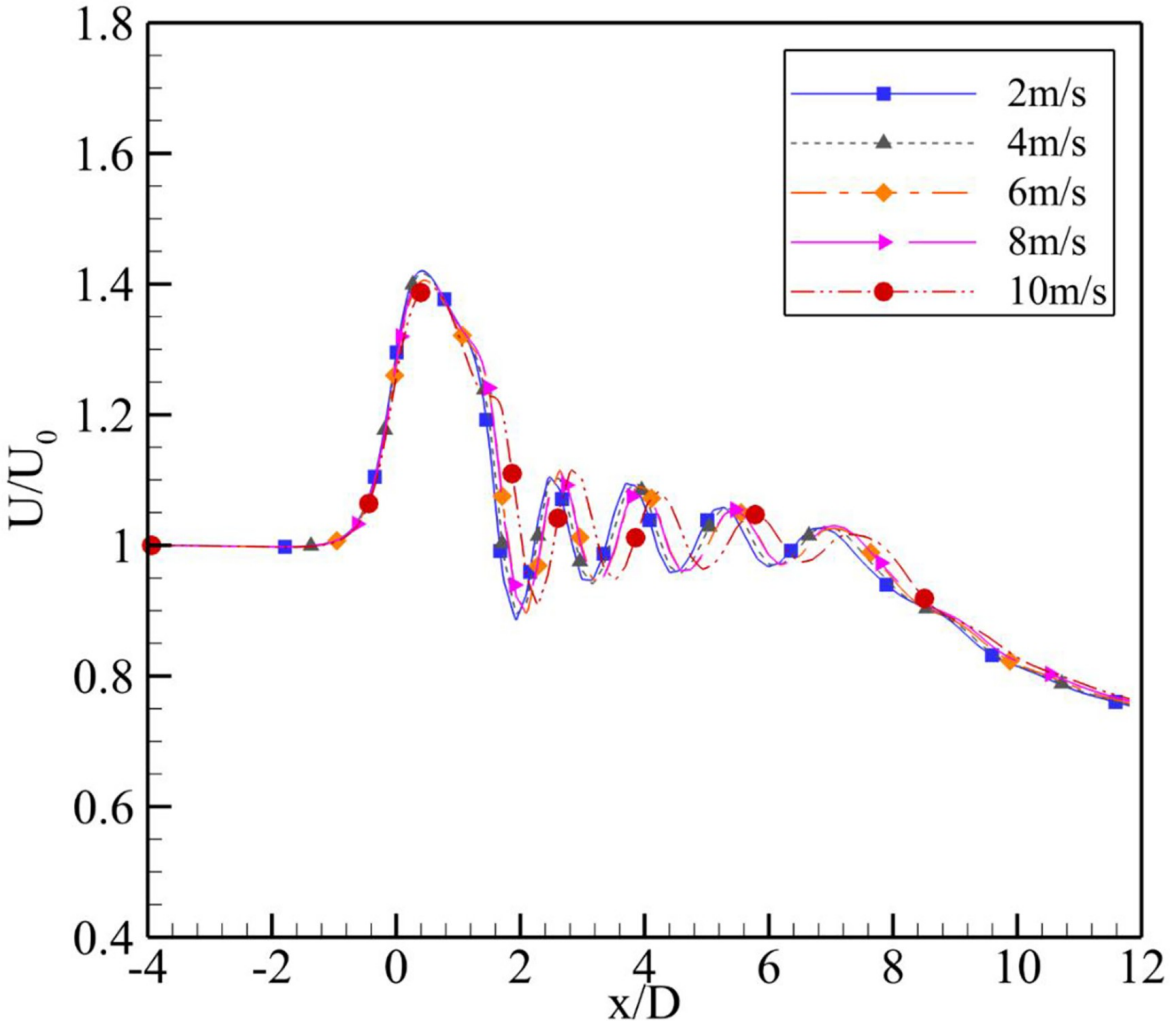
Velocity augmentation at α = 2⁰.

**Fig 7:**
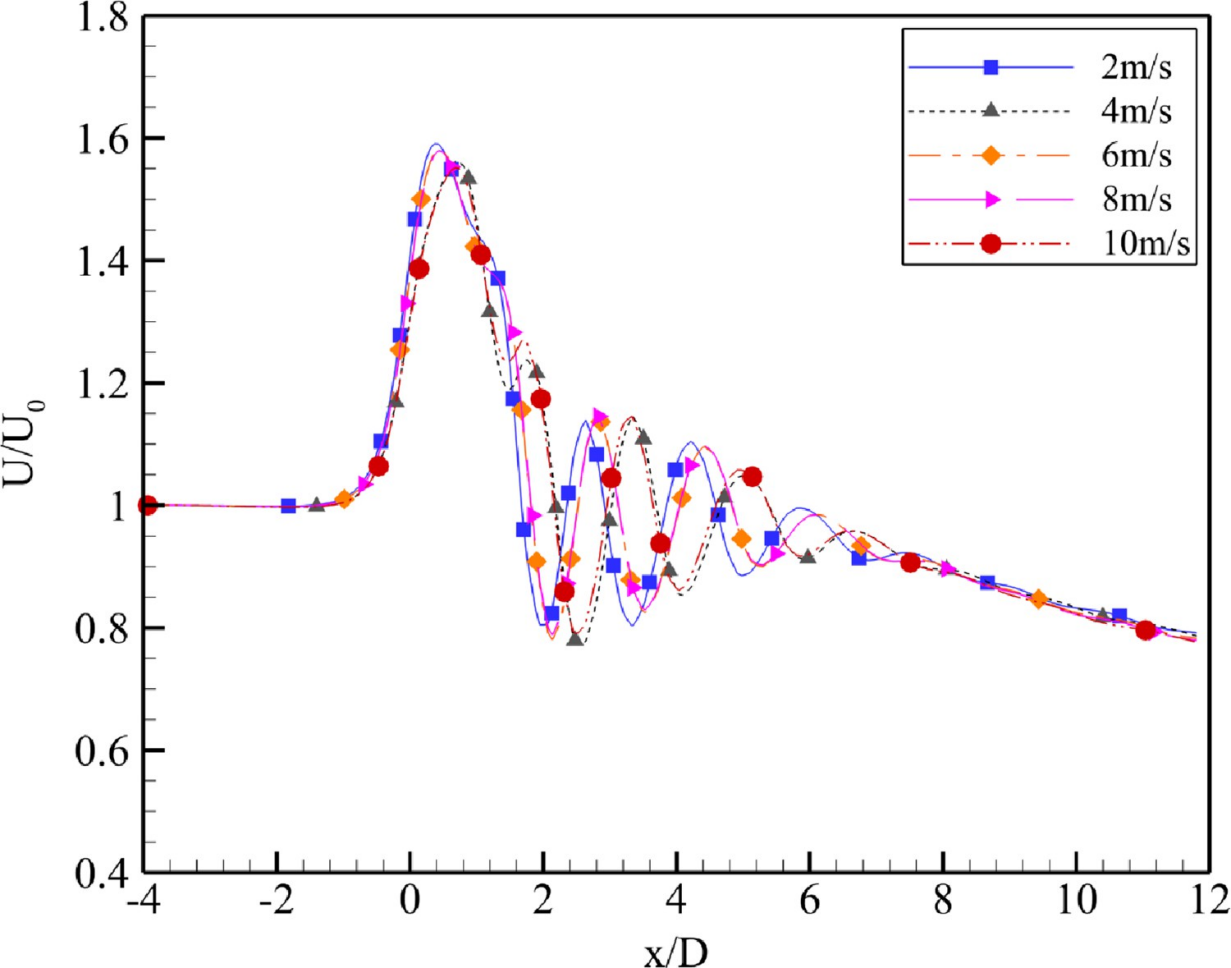
Velocity augmentation at α = 4⁰.

A comparative study at α = 2⁰, 4⁰ and 7⁰ was performed at five different flow speeds other than the optimized values as shown in Figs 5–7. The characteristic curve formed for 2⁰ divergence angle is similar to found by El-Zahaby et al. [33]. The comparison with Abe and Ohya’s experimental work [34] is shown in Fig 5. There is a close agreement with experimental work.

**Fig 8:**
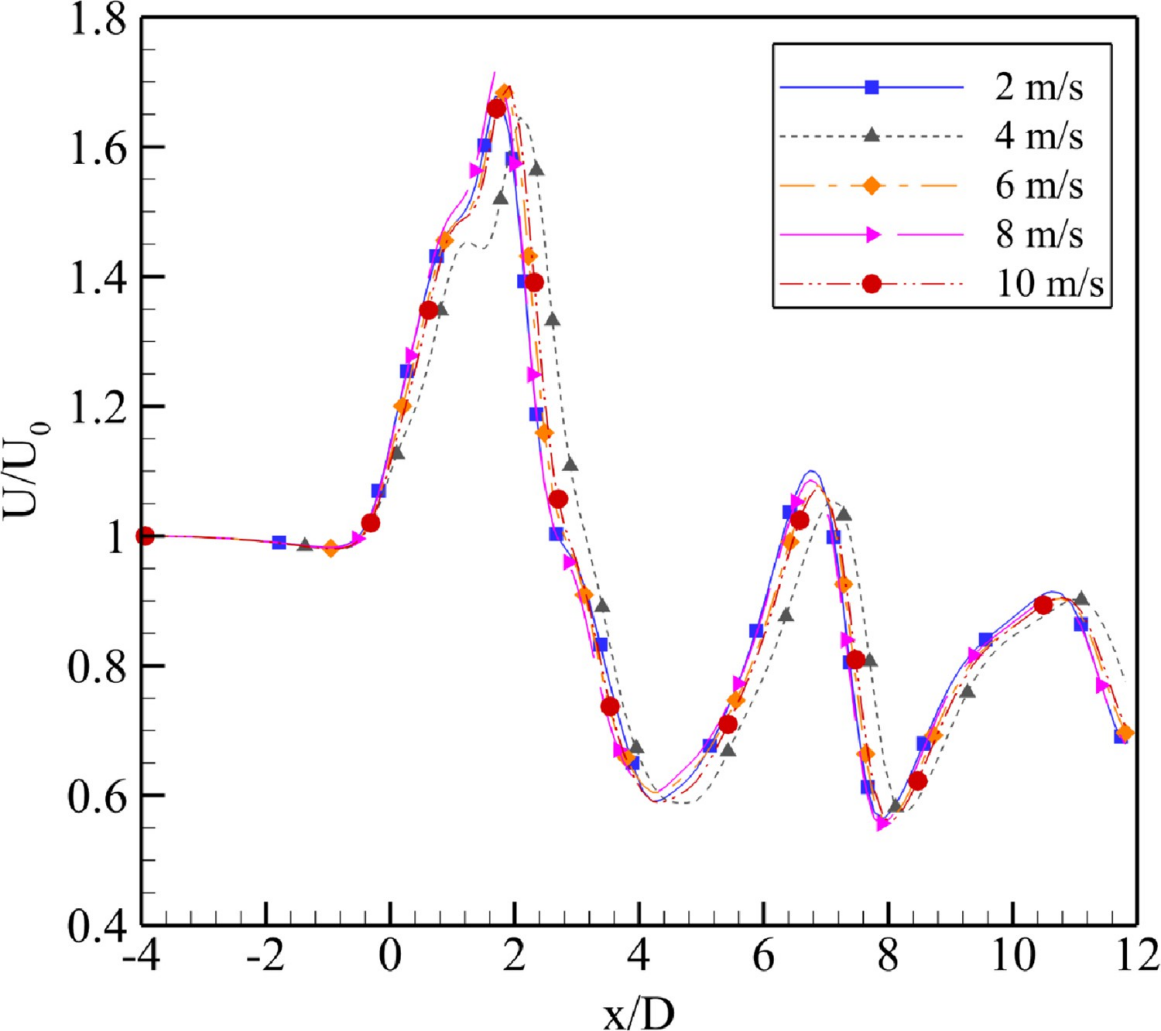
Velocity augmentation at α = 7⁰.

The simulation results reported that the maximum wind energy augmentation was achieved using a 4⁰ degree divergence angle at the actual throat of the diffuser, whereas the minimum was achieved using a 7⁰ divergence angle and the 2⁰ case was intermediary. U_0_ varied from 2 m/s to 10 m/s. From the Fig 6 for α = 2⁰, the throat formed by the fluid remained close to the geometric throat of the diffuser, however, on increasing the U_0_, the throat region moves little forward while decreasing the value of U/U_0_. Similar behavior is observed in Fig 7 for α = 4⁰. The maximum velocity regime shift was for U_0_ = 4 m/s and U_0_ = 10 m/s. These results are in Fig 8 for α = 7⁰. It is evident from the plot that as the U_0_ is increased, the maximum augmentation region moves towards the diffuser from the tail side; however, it will not reach the diffuser throat. The power augmentations obtained show similar results to that reported by [14], however their divergence angles were different.

This behavior is reported by the U_0_ = 8 m/s curve for which the actual throat region formed by the fluid domain moves nearest to the tail end of the diffuser on further increasing the U_0_ to 10 m/s the maximum augmentation region moves to meet the region formed by the U_0_ = 6 m/s.

These results are presented in Fig 8 for the α = 7⁰. It is evident from the plot that as the inlet velocity, U_0_ is increased, the maximum augmentation region moves towards the diffuser from the tail side; however, it will not reach the diffuser throat. This behavior is reported by the U_0_ = 8 m/s curve for which the actual throat region formed by the fluid domain moves nearest to the tail end of the diffuser on further increasing the U_0_ to 10 m/s the maximum augmentation region moves to meet the region formed by the U_0_ = 6 m/s.

Upon increasing the U_0_ for α = 4⁰ as shown in Fig 7, the maximum velocity augmentation region first moved away from the geometric throat and then moved back towards it. The throat region remained close for the inlet velocities of U_0_ = 2, 6 and 8 m/s and moved away for the U_0_ = 4 and 10 m/s. Thus, the maximum velocity augmentation values comparatively decreased for the U_0_ = 4 and 10 m/s.

**Fig 9:**
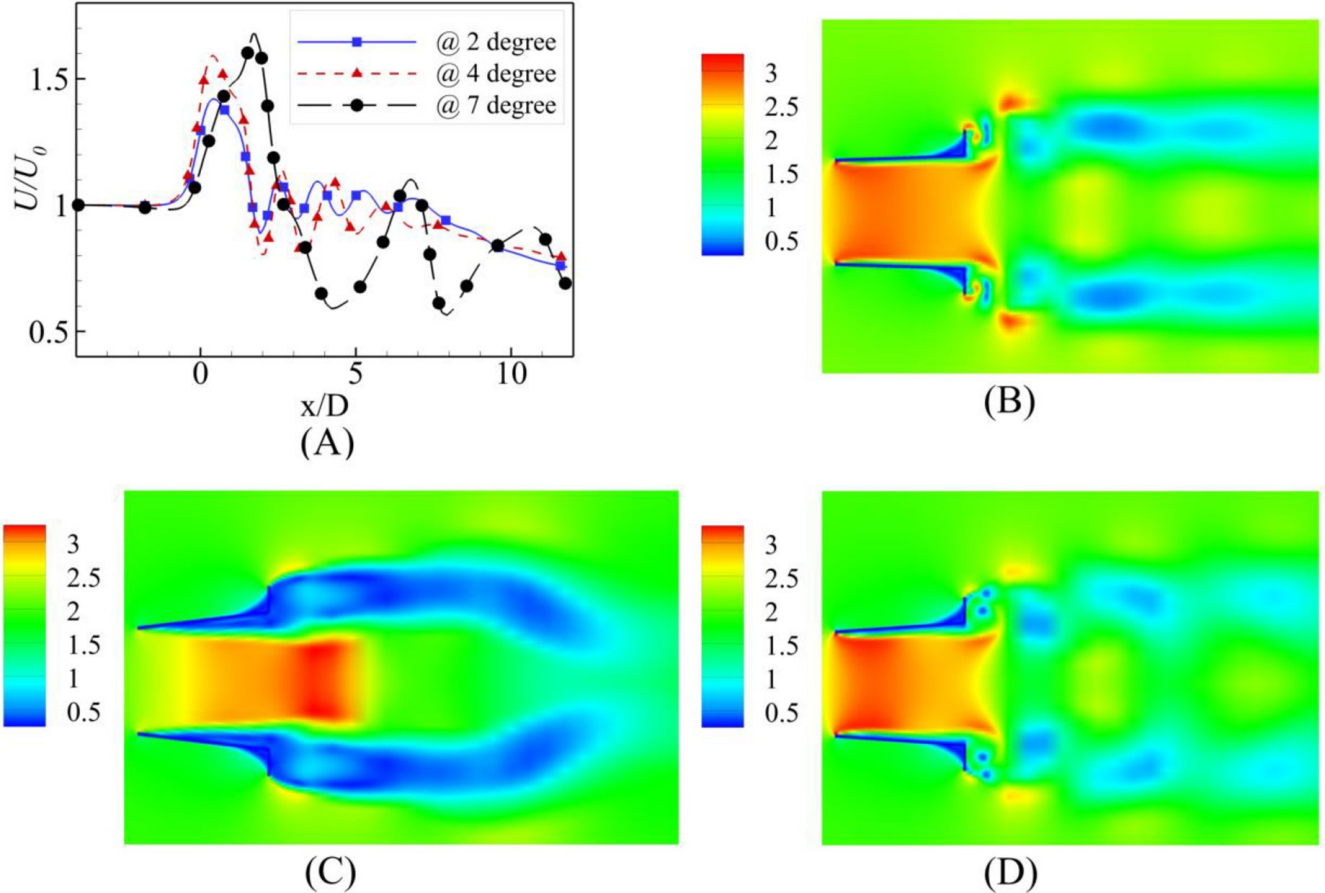
**(A)** Velocity Comparison at U_0_ = 2 m/s; Contours of velocity at α = **(B)** 2⁰ **(C)** 4⁰ **(D)** 7⁰.

**Fig 10:**
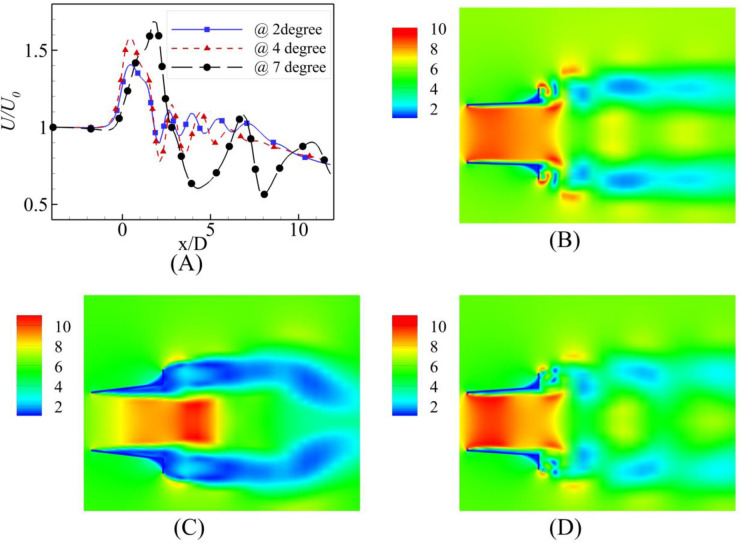
**(A)** Velocity Comparison at U_0_ = 6 m/s; Contours of velocity at α = **(B)** 2⁰ **(C)** 4⁰ **(D)** 7⁰.

**Fig 11:**
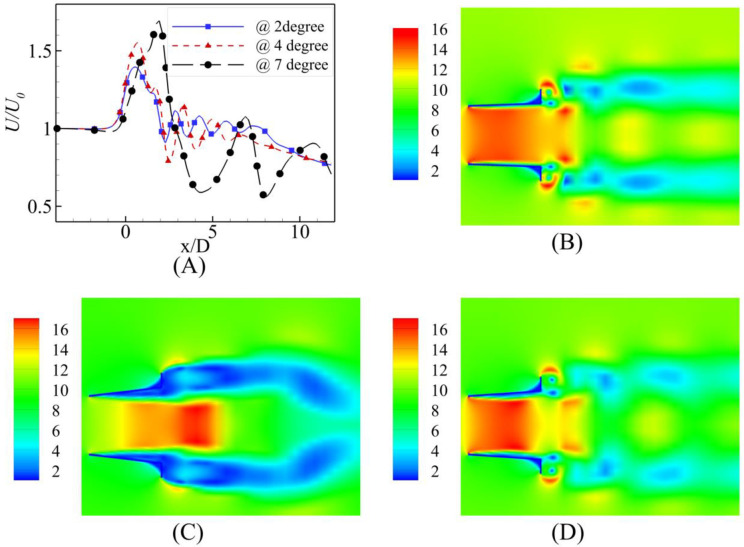
**(A)** Velocity Comparison at U_0_ = 10 m/s; Contours of velocity at α = **(B)** 2⁰ **(C)** 4⁰ **(D)** 7⁰.

The comparisons of 2, 6 and 10 m/s for all divergence angles are shown in Figs 9–11. The graph between *U*/*U*_0_ and *x*/*D* shows the maximum augmentation is achieved at the α = 7⁰ at the tail end of the diffuser. The greater α at constant h caused flow circulations, leading to a further decrease in the throat area away from the diffuser at the downwind side as shown in Fig 9 for U_0_ = 2 m/s.

Comparing the results for higher inlet velocity, U_0_ = 6 m/s at the three different angles, shows similar trends for the α = 2⁰ and 4⁰ to the U_0_ = 2 m/s shown in Fig 10. However, an interesting phenomenon was observed in the case of the α = 7⁰. The throat region formed at the far tail end of the diffuser moved to the near end of the diffuser. Thus, the velocity augmentation was not valuable like the case of the U_0_ = 2 m/s; on the contrary, for the α = 4⁰ and α = 2⁰, the fluid throat was formed at the actual throat of the diffuser.

The graphs and contours for U_0_ = 10 m/s shown in Fig 11 still followed the U_0_ = m/s; however, the maximum velocity augmentation shown for the α = 7⁰ moved closer to the diffuser’s tail end, but it was still out of the diffuser.

**Fig 12:**
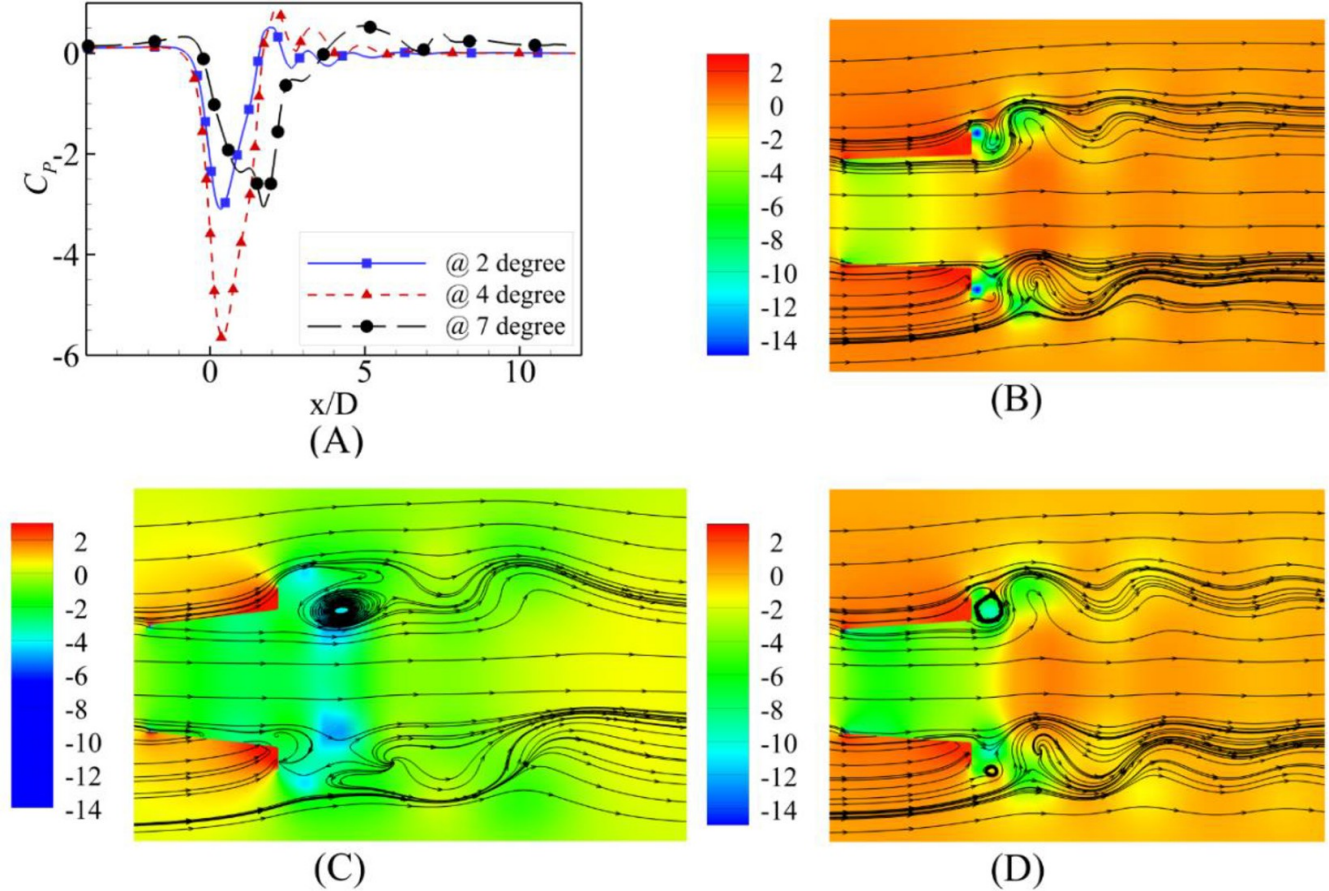
**(A)** Pressure Coefficient at U_0_ = 2 m/s; Contours of velocity at α = **(B)** 2⁰ **(C)** 4⁰ **(D)** 7⁰.

**Fig 13:**
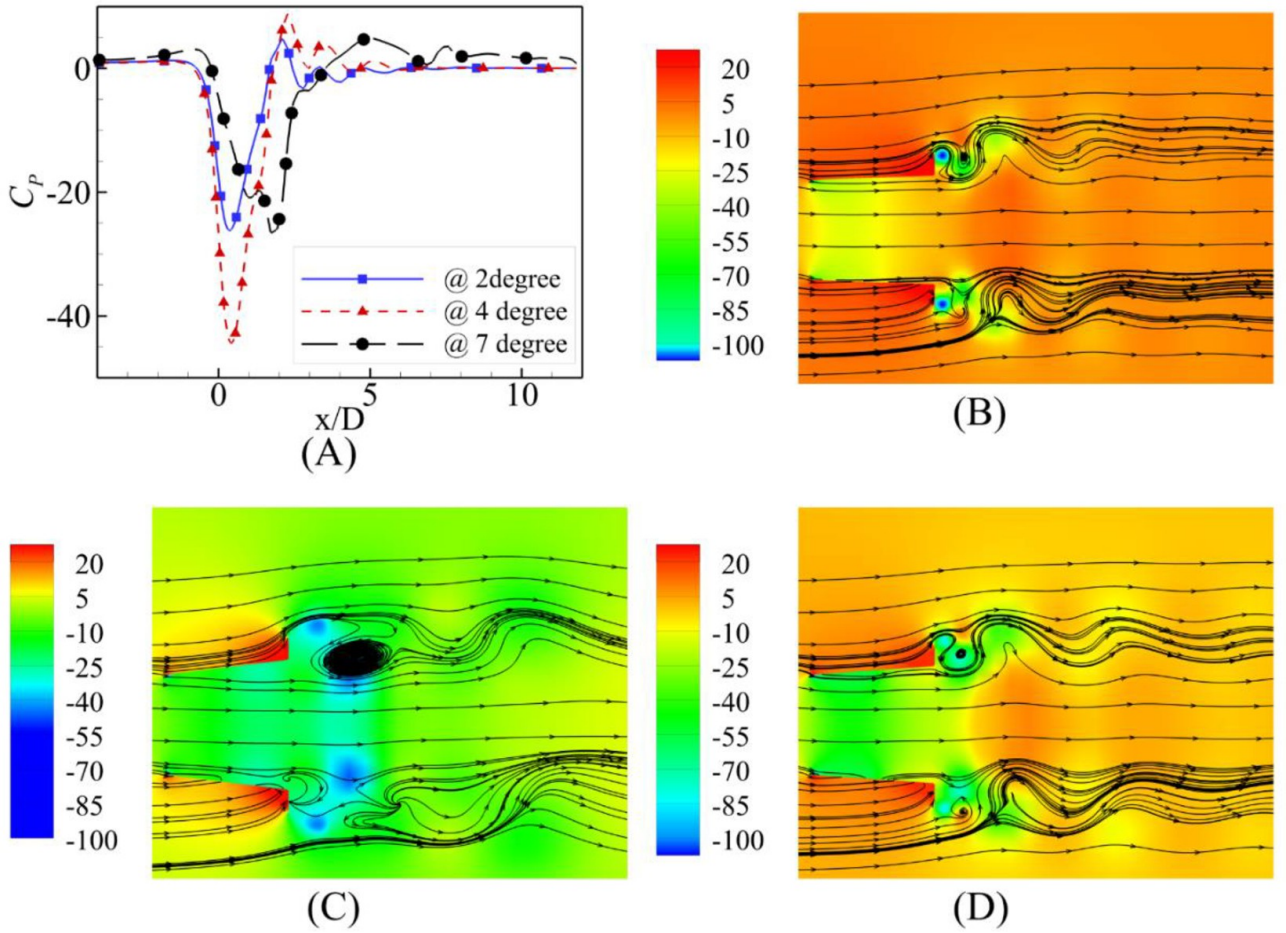
**(A)** Pressure Coefficient Comparison at U_0_ = 6 m/s; Contours of velocity at α = **(B)** 2⁰ **(C)** 4⁰ **(D)** 7⁰.

**Fig 14:**
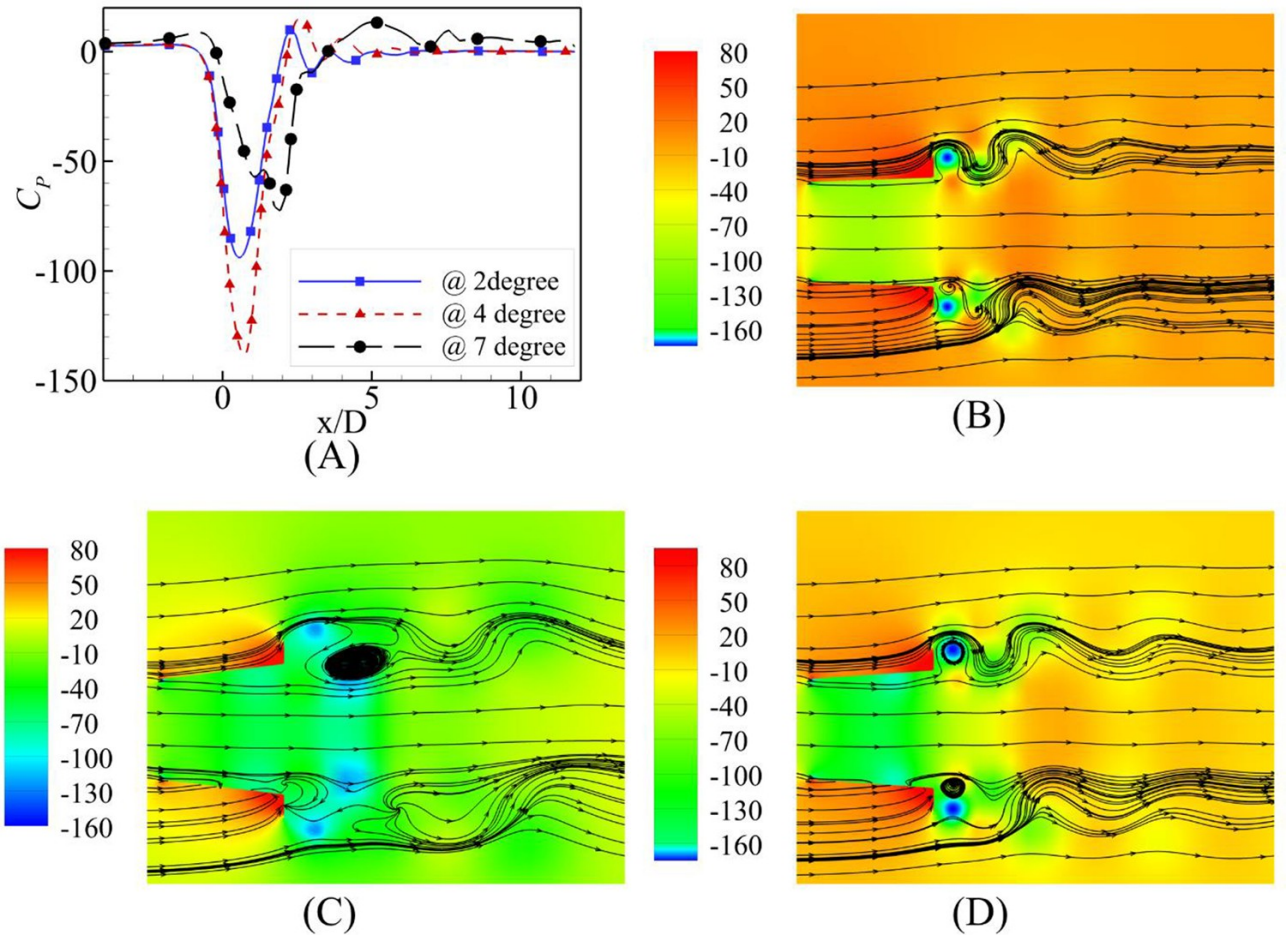
**(A)** Pressure Coefficient Comparison at U_0_ = 10 m/s; Contours of velocity at α = **(B)** 2⁰ **(C)** 4⁰ **(D)** 7⁰.

The flow speed-dependent behavior is evident from the contours of the pressure coefficient and streamlines along with the plot of the pressure coefficient at the central axis shown in Figs 12–14 for U_0_ = 2 m/s, 6 m/s and 10 m/s, respectively. U_0_ = 2m/s is the highest velocity augmentation reported by the α = 7⁰ case. The diffuser achieved the most negative value of the pressure coefficient at the α = 4⁰ divergence angle at the diffuser axis. This difference of augmentation may be due to the consistency of the negative pressure coefficients along a longer length than the α = 2⁰ and α = 4⁰. Visualizing the streamlines on the pressure coefficient contours reports the availability of the flow area was maximum for the α = 2⁰ and flow circulations were just behind the flange for the α = 2⁰ and α = 4⁰.

The investigation of pressure coefficients and streamlines for the U_0_ = 6 m/s and U_0_ = 10 m/s free stream velocities show similar trends; however, the most negative value of the pressure coefficient moves away from the geometric throat of the diffuser for α = 7⁰.

The turbine blades should be located at the maximum velocity augmentation region to maximize benefit of the flanged diffuser. From the above discussion, the location of the wind turbine for maximum power augmentation is not at the geometric throat of the diffuser, but somewhat its location varies concerning U_0_ and α.

### Effect of free-stream velocity on turbine’s location

**Fig 15:**
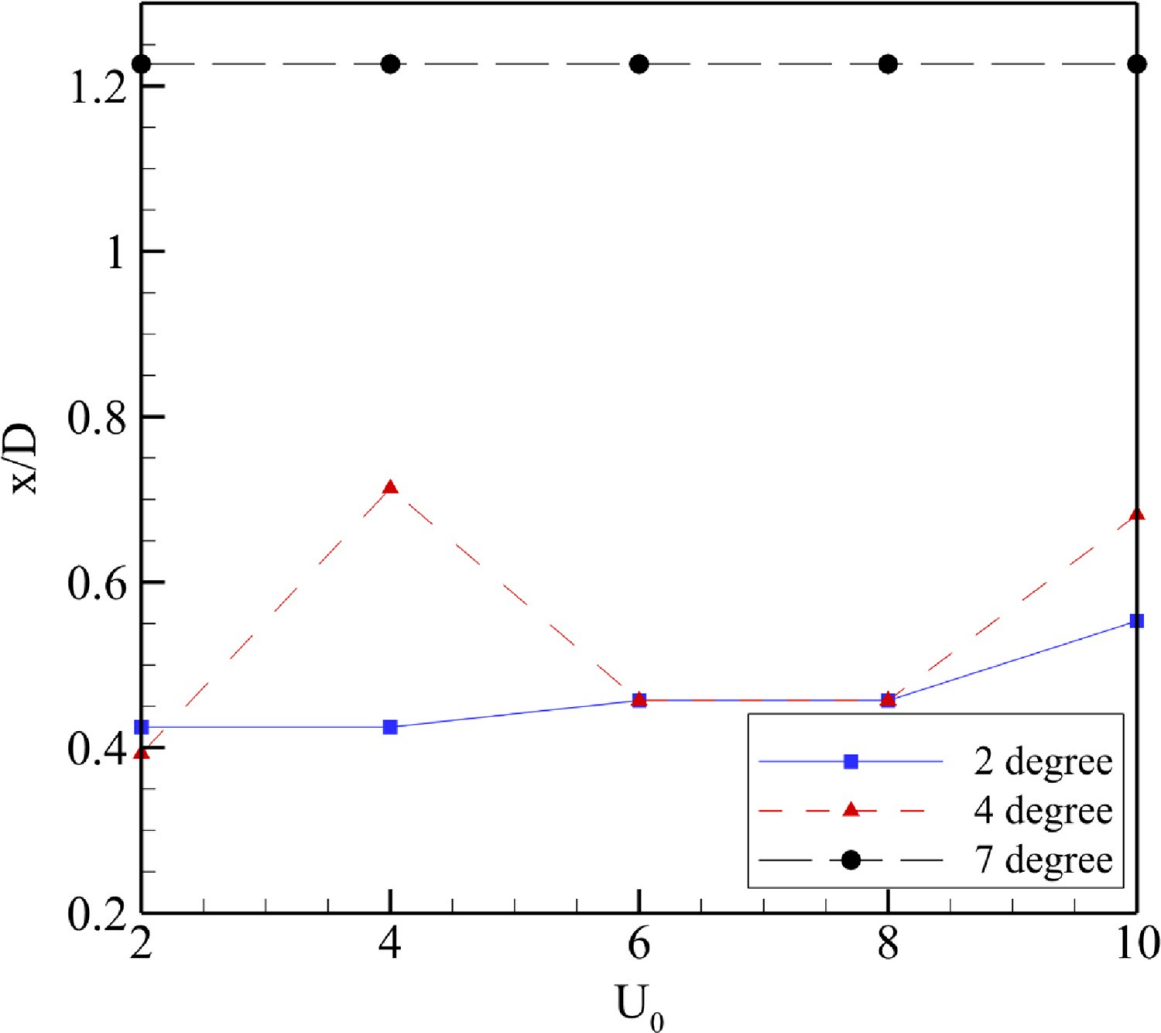
Location of turbine based on U_0_.

In Fig 15, U_0_ has been plotted again x/D averaged over the first five points of maximum velocity augmentation. The general trend for all the velocities showed that the turbine’s location should be shifted further into the diffuser with an increase in velocity. Thus, moving away from the actual geometric throat. The reason could be that with an increase in velocity, there was an increase in vortices formed within and outside the diffuser forcing the maximum augmentation region further into the diffuser. For α = 7⁰, the maximum augmentation location for each free-stream velocity is the same because the fluid throat moved out from the tail end of the diffuser. The graph shows only those values within the diffuser because the turbine should be placed within the diffuser.

### Effect of divergence angle on turbine’s location

**Fig 16:**
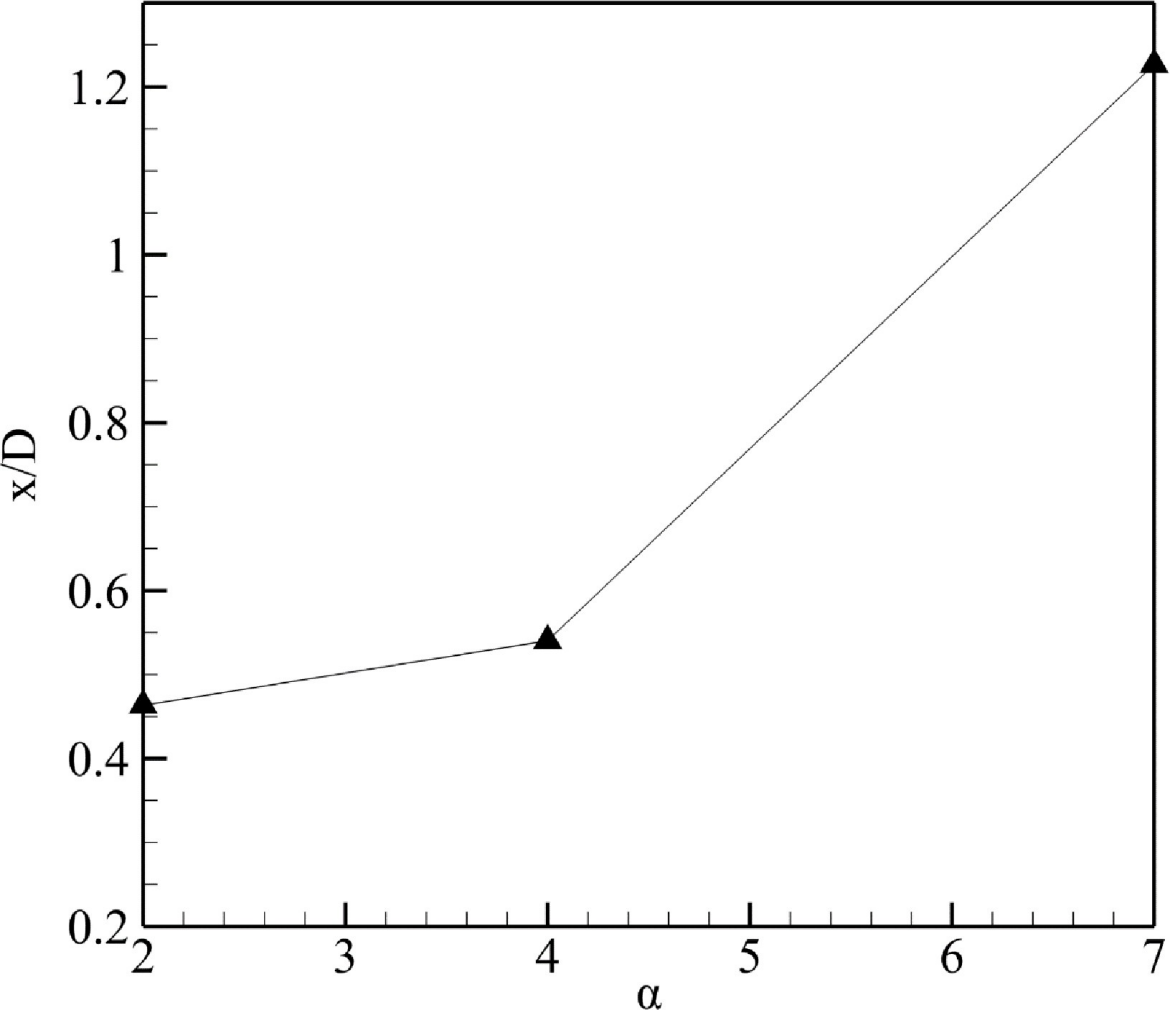
Location of turbine based on α.

With an increase in α, the diffuser wall behaves like a flat plate and leads to flow circulations. The minimum circulations were observed for α = 2⁰, and maximum were observed for α = 7⁰. Fig 16 represents the location of maximum velocity augmentation averaged over the five velocities versus the three divergence angles. From Fig 16, with an increase in α, the wind turbine placement is affected adversely. The turbine moves toward the farther end of the diffuser, thus moving away from the geometric throat.

## Conclusion

In this paper, the diffuser’s divergence angle is numerically investigated to find its effects on the throat velocity and how it affects the location of maximum augmentation point for turbine placement. The maximum velocity augmentation is achieved for the α = 7⁰ with the minimum at α = 2⁰ for the same h optimized for α = 4⁰. The maximum velocity augmentation region formed at the geometric throat of the diffuser as opposed to the actual throat region formed by the fluid away from the geometric throat. The velocity augmentation remains consistent over a range of the free stream flow velocities; however, it varies with free-stream velocity. For α = 2⁰, the variation was slight as compared to α = 4⁰. The diffuser effectiveness for low free stream velocities is evident from the graphs for U_0_ = 2 m/s. Moreover, it was observed that the turbine should be located at 35.6% of diffuser length for α = 2⁰ and this value increased to 36.6% for α = 4⁰ but for α = 7⁰, this value was 94.3%.

In the future, the investigation of the wind turbine’s hub should be included in the numerical investigation of the diffuser’s geometry so that the effect of the tubular channel can be visualized. The optimization parameters should focus on a range of flow velocities to account for the variations in the wind speeds. The location of the throat formed by the fluid regime should be carefully investigated to achieve maximum power augmentation at the geometric throat for the flange augmented diffuser for a horizontal axis wind turbine.

## Abbreviations

D: Rotor’s Diameter, m
h: flange height, m
α: divergence angle, degree (°)
x: coordinate, m
y: coordinate, m
u: non-dimensional velocity in *x*
v: non-dimensional velocity in y
P: non-dimensional static pressure
t: non-dimensional time
U: Local Velocity of wind, m/s
P: Power, W
ⴄ: Efficiency
Ω: Ohms
ρ: density, kg/m^3^
A: Area of cross-section, m^2^
Re: Reynolds number
Y^+^: Y-plus value
0: free stream velocity
throat_avg: average value at throat
exp: Experimental value
num: Numerical value
CFD: Computational Fluid Dynamics
HAWT: Horizontal Axis Wind Turbine
DAWT: Diffuser Augmented Wind Turbine
